# Regional brain aging: premature aging of the domain general system predicts aphasia severity

**DOI:** 10.1038/s42003-024-06211-8

**Published:** 2024-06-11

**Authors:** Natalie Busby, Sarah Newman-Norlund, Sara Sayers, Chris Rorden, Roger Newman-Norlund, Janina Wilmskoetter, Rebecca Roth, Sarah Wilson, Deena Schwen-Blackett, Sigfus Kristinsson, Alex Teghipco, Julius Fridriksson, Leonardo Bonilha

**Affiliations:** 1 Department of Communication Sciences and Disorders, University of South Carolina, Columbia, SC USA; 2 Department of Psychology, University of South Carolina, Columbia, SC USA; 3 Department of Health and Rehabilitation Sciences, Medical University of South Carolina, Charleston, SC USA; 4 Department of Neurology, Emory University, Atlanta, GA USA; 5 School of Medicine, University of South Carolina, Columbia, SC USA

**Keywords:** Neural ageing, Cognitive ageing

## Abstract

Premature brain aging is associated with poorer cognitive reserve and lower resilience to injury. When there are focal brain lesions, brain regions may age at different rates within the same individual. Therefore, we hypothesize that reduced gray matter volume within specific brain systems commonly associated with language recovery may be important for long-term aphasia severity. Here we show that individuals with stroke aphasia have a premature brain aging in intact regions of the lesioned hemisphere. In left domain-general regions, premature brain aging, gray matter volume, lesion volume and age were all significant predictors of aphasia severity. Increased brain age following a stroke is driven by the lesioned hemisphere. The relationship between brain age in left domain-general regions and aphasia severity suggests that degradation is possible to specific brain regions and isolated aging matters for behavior.

## Abstract (web summary)

Individuals with stroke aphasia have premature brain aging in intact regions of the lesioned hemisphere. In left domain-general areas, premature brain aging significantly predicts aphasia severity, suggesting that isolate aging matters for behavior.

## Introduction

Older age is associated with cognitive decline^1–3^. Increased age is also associated with a higher incidence of strokes and worse recovery following a stroke^4–7^. Aphasia is a common neurological sequela following a left-hemisphere stroke and older individuals are more likely to have severe aphasia immediately following a stroke^6^ and long-term severe language deficits^4^.

Despite the overall, group level relationship between cognition, stroke recovery, and age, there are large interindividual differences in age-related cognitive decline and recovery trajectories following a stroke^8–10^. This is commonly recognized in the clinical setting, and the concept of cognitive reserve is often postulated as the potential mechanism underlying variable personalized clinical trajectories. Cognitive reserve is grossly defined as the capacity to preserve cognition despite aging or the increased resilience to neurological injury^11,12^. A potential marker for cognitive reserve is the preservation of structural brain integrity, which is defined as the rate of gray matter atrophy. Indeed, whole brain volume decline is associated with worsening cognitive skills such as working memory or processing speed^1–3^. Gray matter atrophy is also a possible contributing factor to the relationship between increased age and poorer recovery following a stroke, with the integrity of the spared brain tissue being a marker for cognitive reserve and the capacity for recovery^13–17^.

Indeed, gray matter integrity across individuals of the same chronological age can vary substantially, with some individuals exhibiting gray matter decline at levels observed in much older individuals and others exhibit preserved brain structure well into advanced age^18^. Whereas whole brain volume can be used as a personalized measure of brain integrity, recent studies have demonstrated that age-related brain changes can be better estimated by assessing the weighted distributed atrophy of brain regions, since some regions can exhibit more atrophy compared with others^19,20^. For example, Hedden and Gabrieli (2004) found that the largest age-related volumetric changes occur in the prefrontal cortex whereas pathological aging, which they define as those which accompany Alzheimer’s disease, typically affect hippocampal regions^1^. Similarly, regional atrophy differences have been found following a stroke, with ref. ^21^. finding reductions in ipsilateral thalamic volume in the first three months post-stroke^21^, and Seghier and colleagues finding accelerated atrophy across the whole ipsilateral hemisphere in the years following a stroke^22^.

This new field of research leverages quantitative neuroimaging to determine age-like changes in brain or other tissues to measure the observed effects on aging at the organ level. Thus, the novel concept of brain age has recently emerged in neurology and neurosciences, and it is becoming increasingly recognized as a marker for cognition and cognitive reserve. Brain age is estimated at the individual level by comparing regional gray matter tissue volumes from one individual against a normative database of healthy individuals^15,23^. Brain age calculation depends on multivariate models, where atrophy (i.e., reduced gray matter volume) of different brain regions has a distinct weight as a predictor of age-related decline, the result is an estimate of the expected age of the individual based on the integrated brain tissue information.

The difference between an individual’s chronological age and the estimated brain age provides information about the health of the brain tissue relative to typical brain aging. Premature brain aging (defined as higher estimated brain age compared with chronological age) is an important predictor of lower cognition among older adults in general^12,24^. Premature brain aging is associated with poorer cognitive reserve and lower resilience to injury^15^.

Importantly, strokes are associated with advanced brain age likely due to a multifactorial phenomenon. That is, many stroke survivors have at least one cardiovascular risk factor, which are independently associated with higher brain age. However, the stroke lesion may also be independently associated with decline in brain integrity and advanced brain age. For example, stroke survivors typically have a higher brain age compared to age-matched controls when measured as early as six weeks post-stroke and this difference remains one-year post-stroke^25^. However, increased brain age may be a consequence of the stroke, or a pre-morbid factor associated with an increased risk of stroke^25^. Nonetheless, among individuals with aphasia, increased brain age (in the acute and chronic stages) is associated with more severe aphasia^15^.

Notably, brain aging is a concept that has been largely defined based on healthy individuals without focal brain lesions. It is however possible that focal brain lesions (e.g., strokes) are associated with differential levels of brain aging within the same person, as focal injury may lead to a directed injury to a group of regions or to a neuroanatomical system. More specifically, some regions of the brain may age faster than others^26^. Accelerated brain aging in certain regions of the brain such as temporal gray matter has been associated with the development of mild cognitive impairment^27^. In stoke, injury from disconnection or from reduced cognitive engagement may be associated with worse progressive atrophy and regional aging. Importantly, we hypothesize that atrophy (i.e., reduced gray matter volume) within specific brain systems commonly associated with language recovery may be important determinants of variance in long-term language deficits, controlling for the stroke lesion, chronological age, or non-specific brain atrophy (i.e., gray matter volume). In this study, we tested our hypothesis by applying a brain age method to estimate global and regional brain age based on a large normative cohort of healthy controls, which was used to investigate the relationship between regional brain aging and aphasia severity.

## Results

### Stroke participants

**Fig. 1:**
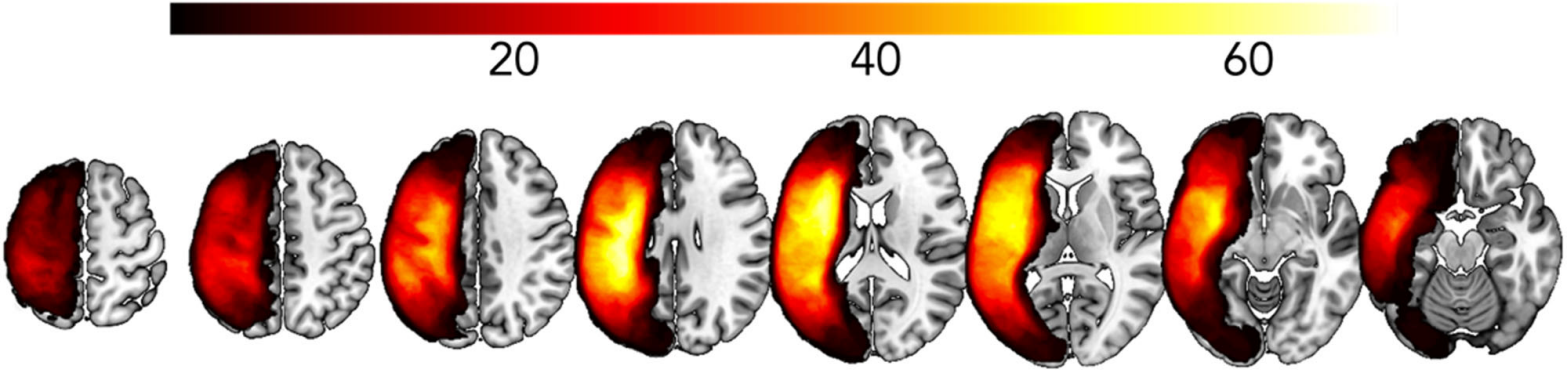
Lesion overlay for participants with aphasia (*n* = 89). Lesion overlay where brighter yellow regions indicate more participants have a lesion, and darker red and black regions indicate that less people have a lesion.

See Fig. 1 for lesion overlay of all stroke participants. Demographic information describing these participants are provided in Supplementary Table 1. Stroke participants were, on average, 60.60 years (SD = 11.27 years) which was significantly older than within-range (*t* = −7.082, *p* < 0.001) and out-of-range controls (*t* = 7.381, *p* < 0.001). Participants had an average of 15.49 years of educations (SD = 2.28) which was significantly less than within-range controls (*t* = 3.079, *p* = 0.002) but there was no significant difference compared to out-of-range controls (*t* = 0.229, *p* = 0.820). There were more males than females (males: 60.67%, females: 39.33%) which was significantly different to within-range controls (males: 29.37%, females: 70.63%, *t* = −4.274, *p* < 0.001) and out-of-range controls (males: 82.08%, females: 17.92%, *t* = −6.262, *p* < 0.001). Finally, the majority of participants with stroke were right-handed, (left-handed: 10.23%, right-handed: 89.77%) which was not significantly different to within-range controls (left-handed: 6.35%, right-handed = 89.68%, ambidextrous = 3.97%, *t* = 1.629, *p* = 0.105) but was significantly different to out-of-range controls (4.7% left-handed, 91.5% right-handed, 3.77% ambidextrous, *t* = 1.984, *p* = 0.048). See Supplementary Table 1 for a full list of demographics for stroke participants, and Supplementary Table 2 for control participant demographics.

### Healthy controls

Data from healthy controls with an estimated brain age within 5% of their chronological brain age (i.e., within-range controls, *n* = 126) were entered into linear models. Demographic information describing these participants, along with those excluded at this stage (i.e., out-of-range controls, *n* = 106) are provided in Supplementary Table 2.

### Mixed effects ANCOVA between hemispheres and controls vs stroke participants

**Fig. 2:**
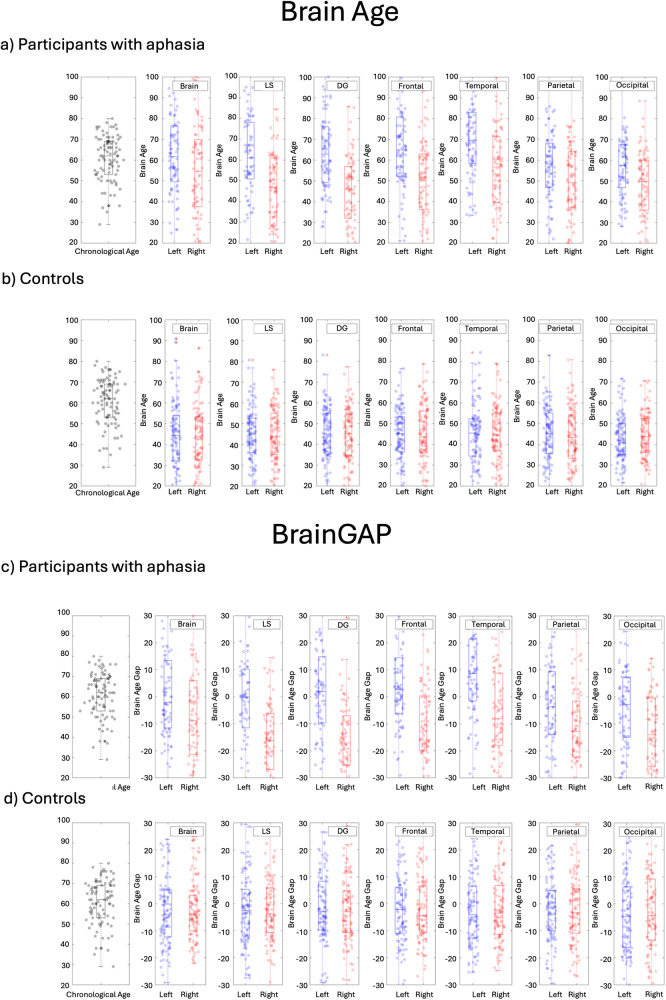
Box plots showing chronological age alongside brain age and BrainGAP. Box plots showing chronological age (black dots) then brain age (**a**, **b**) and BrainGAP (**c**, **d**) for left (blue dots) and right (red dots) hemisphere in the following brain regions: whole brain, language-specific regions, domain-general regions, frontal regions, temporal regions, parietal regions, and occipital regions. **a**, **c** show participants with aphasia, (**b**, **d**) show out-of-range controls. Note that graphs showing control participants include only out-of-range controls (i.e., control participants who had a brain age >5% higher or lower than their chronological age). LS language-specific, DG domain-general.

The two-way mixed-effects ANCOVA within the domain general regions revealed that there was no significant main effect of group (out-of-range control vs. stroke aphasia), *F (*1,263) = 0.039, *p* = 0.844. Age and education were significant predictors (*F*(1,263) = 45.300, *p* < 0.001, and *F*(1,263) = 12.259, *p* < 0.001 respectively). There was also a significant interaction between hemisphere and group, *F* (1,263) = 18.178, *p* < 0.001, where participants with stroke aphasia had an increased regional brain age in the left hemisphere ROIs compared to out-of-range controls, see Fig. 2. It is important to note that these analyses and Fig. 2 show only out-of-range controls (i.e., control participants who had a whole brain age of >5% higher or lower than chronological age). Regional brain age could not be computed for in-range controls because they were used to create the model used to estimate brain age.

### Independent samples T-Test between controls and stroke participants

Participants with stroke aphasia were older (M = 60.60, *SD* = 11.27) than controls (M = 46.80, *SD* = 17.08, *t*(193) = −6.517, *p* < 0.001). However, the range of both is similar: controls 20−79 years, stroke aphasia 29−80 years.

### Correlational analysis between regional brain age and gray matter volume

**Fig. 3:**
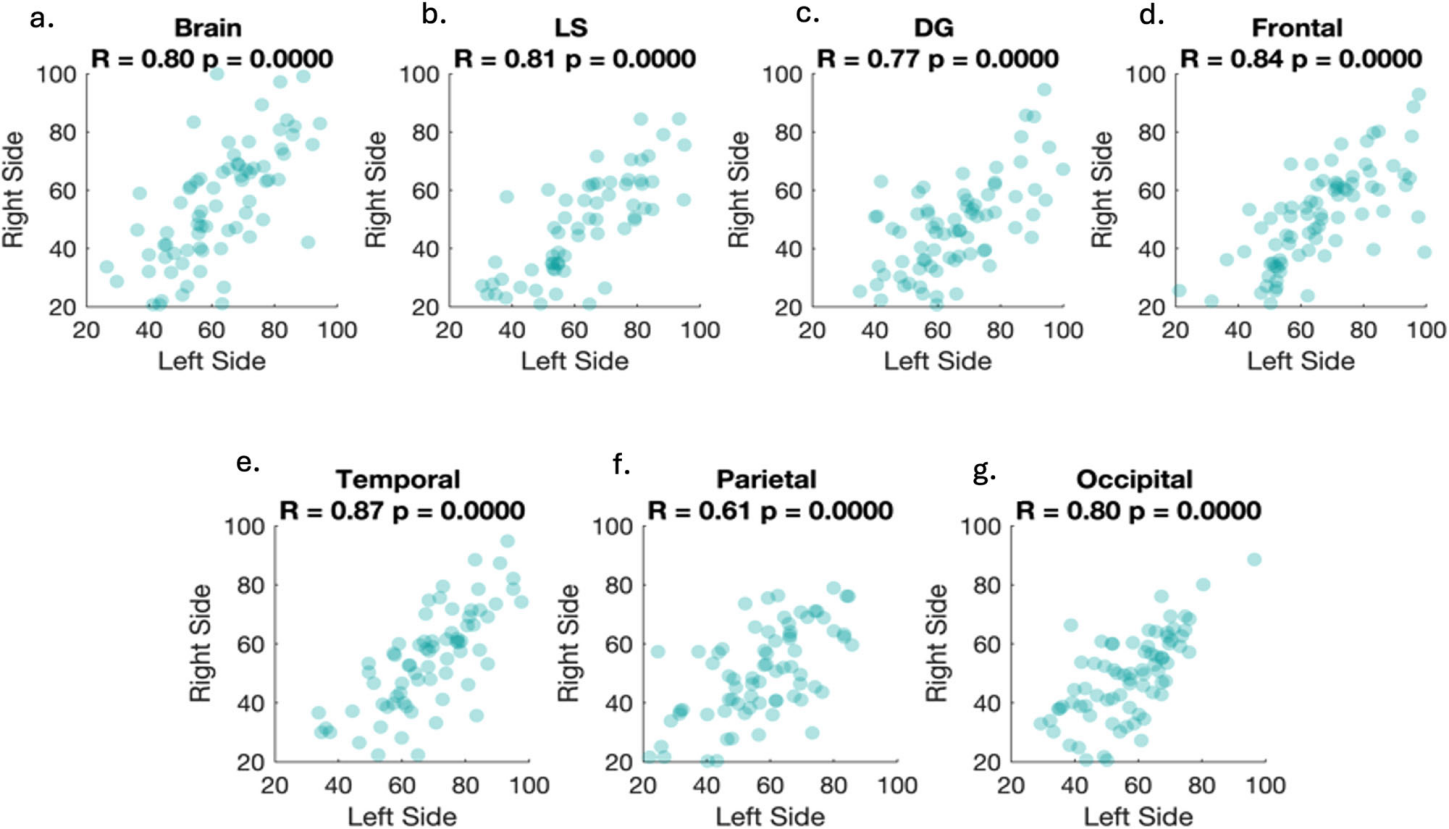
Brain age estimations for participants with aphasia. Scatterplots showing brain age estimations for participants with aphasia across different brain regions: (**a**) whole brain, (**b**) language-specific regions, (**c**) domain-general regions, (**d**) frontal regions, (**e**) temporal regions, (**f**) parietal regions, (**g**) occipital regions.

**Fig. 4:**
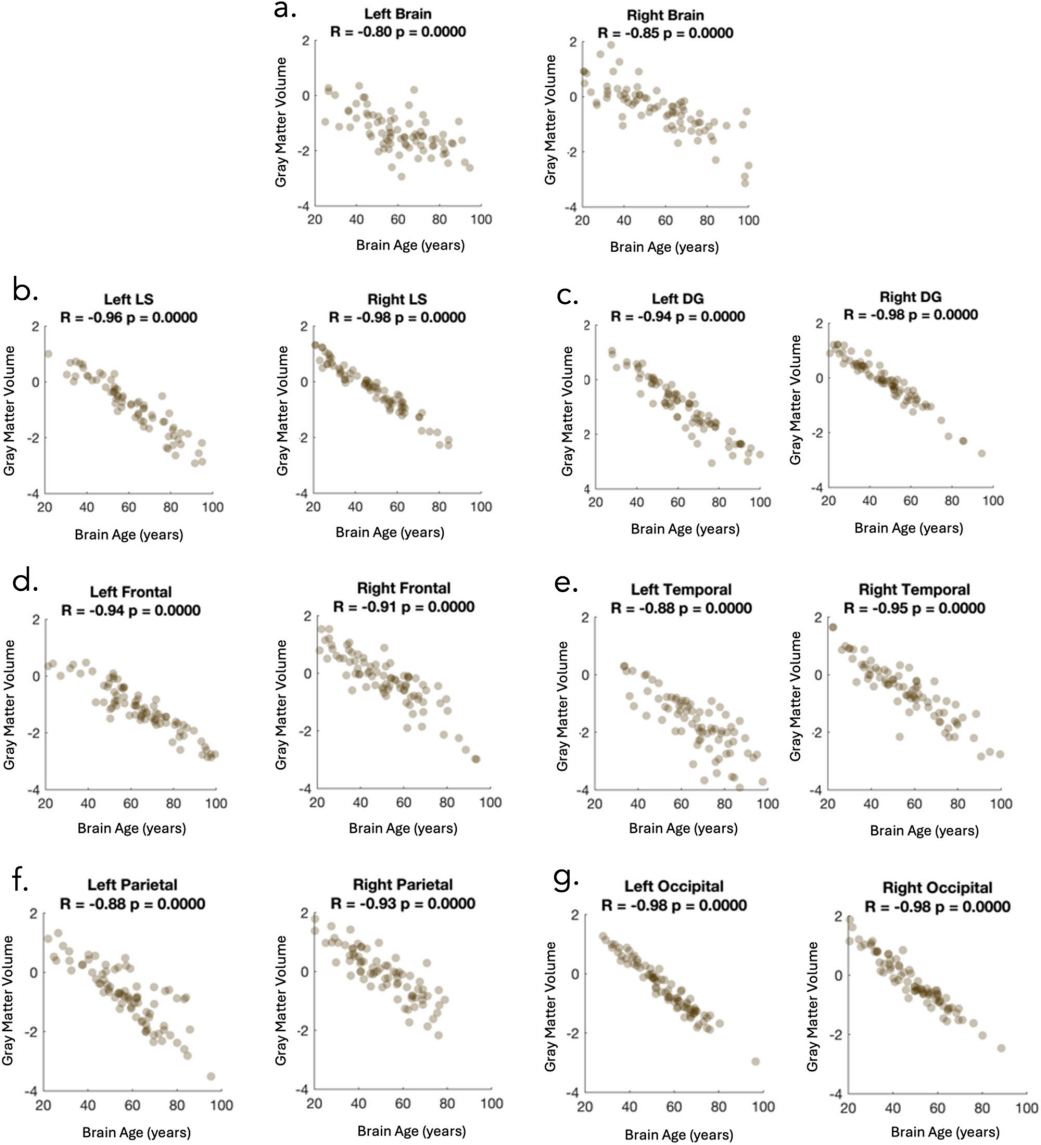
The relationship between gray matter volume and regional brain age. Scatterplots to show the relationship between gray matter volume and regional brain age across all regions. **a** left and right hemispheres, (**b**) left and right language-specific regions, (**c**) left and right domain general regions, (**d**) left and right frontal regions, (**e**) left and right temporal regions, (**f**) left and right parietal regions, and (**g**) left and right occipital regions. Gray matter volume is shown in z-scores. Abbreviations: LS Language specific, DG Domain general.

Pearson correlations between the brain age of the left and right hemisphere were calculated for all regions. A strong positive correlation was found between the left and right hemisphere for all regions (ranging from *r* = 0.61 to 0.87), see Fig. 3. To account for multiple comparisons, we use the adjusted p-value of 0.007 (0.05/7). It should be noted that correlations involving language-specific regions may not be accurate due to the limited number of participants with intact language-specific ROIs. Correlation analysis also revealed a significant negative relationship between regional brain age and regional gray matter volume in both hemispheres in every tested region (ranging from *r* = −0.80 to −0.98), see Fig. 4. We account for multiple comparisons by using an adjusted *p* value (0.05/14 = 0.0036). It is important to note that these two scores (regional brain age and regional gray matter volume) are not exactly the same as calculating regional brain age using the linear regression equation, each ROI contributes differently to the overall brain age estimation (i.e., gray matter volume in some ROIs is very important and gray matter volume in other ROIs may not be as important). However, there would be a perfect correlation between gray matter volume and brain age if we calculated it using only one ROI at a time.

### Number of ROIs used in the linear model

There was variation in the number of ROIs used in the linear model for each stroke participant, depending on which ROIs were lesioned and which were intact. Of the 8 domain-general regions, 3 stroke participants had 0 intact regions so were excluded from this analysis. Of the remaining included participants, the minimum intact ROIs was 1 and the max was 8, with participants having an average of 4.45 intact regions (standard deviation = 2.04, median = 4).

We investigated the dispersion of the residuals to determine whether the number of ROIs used to estimate regional brain age affected the accuracy of the brain age prediction (i.e., if the domain-general regional brain age prediction would be worse for Participant A who had a lesion which covered 6 ROIs in the left domain-general region [i.e., 2 spared ROIs] vs Participant B who had a lesion covering no ROIs in the left domain-general region [i.e., 8 spared ROIs]). For the left domain-general region, there was a negative correlation between the number of ROIs used and the standard deviation of the residuals (*R* = −0.79, *p* < 0.001). However, there was not a significant correlation between the number of ROIs used and the mean of the residuals (*R* = −0.17, *p* = 0.1114). Similarly, for the whole left hemisphere region, there was a negative correlation between the number of ROIs used and the standard deviation of the residuals (*R* = −0.94, *p* < 0.001). However, there was not a significant correlation between the number of ROIs used and the mean of the residuals (*R* = −0.11, *p* = 0.308).

### Multiple linear regression analysis between regional brain age and behavior

Multiple linear regression analysis between left domain-general regions and WAB AQ revealed that BrainGAP (*p* = 0.008), gray matter volume (*p* = 0.009), lesion volume (*p* < 0.001), and age (*p* < 0.001) were significant predictors of WAB AQ. Similarly, BrainGAP (*p* = 0.001), gray matter volume (*p* = 0.001), and lesion volume (*p* < 0.001) were significant predictors of the WAB comprehension subscore, see Supplementary Table 3 for a full breakdown of the results. Number of ROIs used to generate the brain age estimation model was only a significant predictor of the spontaneous speech subtest (*p* = 0.007).

## Discussion

To investigate the relationship between regional brain aging and aphasia severity, we created a model based on healthy participants to estimate regional brain age. Although there was a strong relationship between aging in the left and right hemispheres, for participants with stroke aphasia, estimated brain age was higher for left-sided structures. There was also a strong, but not complete, relationship between BrainGAP and gray matter volume. As the stroke participants in this study had large lesions to language-specific regions in the left hemisphere, we focus on left domain-general regions. Our multiple linear regression analysis revealed that for left domain general regions, BrainGAP, gray matter volume, age, and lesion volume were significant predictors of aphasia severity and language comprehension.

It has been previously reported that following a stroke, individuals typically have an increased brain age compared to chronological age-matched controls^25^. Although the current finding did not find that participants with aphasia had an older brain age across the entire brain, it does extend this notion by demonstrating that the brain does not age in the same way across all regions, particularly if there is a lesion. This is highlighted by our finding that for left hemisphere stroke participants, but not controls, intact left hemisphere regions had an increased brain age (i.e., premature brain aging) compared to right hemisphere homologues, though it is important to note that only out-of-range controls were used for this comparison. By calculating brain age separately for different regions, we were able to demonstrate that the perilesional hemisphere (excluding the lesion) has an increased BrainGAP in participants with aphasia, where individuals with a left hemisphere stroke have an increased brain age in the left hemisphere. One possible explanation for this is that both hemispheres are aging at the same rate, but due to the stroke, the left hemisphere ages faster than the right. An alternative is that prior to the stroke, left hemisphere regions were already affected and may have had an increased brain age. It is possible that we did not find an increased brain age across both hemispheres in stroke participants compared to controls because we used only out-of-range controls for comparisons (i.e., control participants with a brain age >5% higher or lower than chronological age). This was because it was not possible to use within-range controls for this comparison, as they are used in the model to estimate regional brain age.

Following a stroke, the integrity of the spared tissue is likely an important factor influencing recovery trajectories^13–17^. Indeed, research has demonstrated that markers of reduced white matter integrity, such as white matter hyperintensities, have been associated with poorer stroke recovery^4,16,17,28^. Similarly, gray matter atrophy (i.e., reduced gray matter integrity) may be related to recovery trajectories or the capacity for therapy gains^13–17^. Previously, global premature brain aging (higher BrainGAP) has been associated with increased aphasia severity and reduced therapy gains^14,15^. This paper demonstrates that there are different patterns of aging across the brain, and it likely follows that aging in some brain regions may be more important for predicting behavior than others. For example, it may be that, following a stroke to language-specific regions of the brain, individuals may rely on domain general regions to support behavioral recovery. Indeed, the multiple linear regression results reveal that brain aging, specifically in domain general regions is independently associated with overall aphasia severity, as well as specific aspects of language including language comprehension. These results highlight the notion that isolated aging matters for behavior, and degradation to specific brain regions may be associated with behavioral outcomes.

Although in this study regional brain ages are calculated from the gray matter volume, correlations demonstrate that these estimated regional brain age scores are not exactly the same measure as gray matter atrophy (i.e., volume). As expected, there was a strong correlation between the two; however, there was variability in the magnitude of the relationship depending on the brain region (e.g., left hemisphere: *R* = −0.80, left domain general: *R* = −0.94). This suggests that different brain regions contribute differentially to the brain age calculation. For regions with higher correlations with BrainGAP and gray matter volume (e.g., left or right occipital regions [*R* = 0.98]), it is likely that all regions contribute a similar amount to the brain age estimation. However, for regions with slightly weaker correlations (e.g., left hemisphere: *R* = −0.80, left domain general: *R* = −0.94), it is likely that atrophy (i.e., reduced gray matter volume) in some ROIs is more closely associated with chronological age in controls.

This is corroborated in the multiple linear regression results where both BrainGAP scores and atrophy (i.e., reduced gray matter volume) independently predicted all behavioral scores. Again, this highlights that aging to some brain regions may be more important than others. Overall gray matter degradation in older age has previously been associated with health factors such as hypertension or diabetes^29–31^, therefore future research could investigate if specific health or demographic factors affect gray matter volume differently across the brain.

In line with previous research, lesion volume was a predictor of aphasia severity (WAB AQ) as well as all WAB subtest scores except spontaneous speech^4–7^. Similarly, age was a consistent predictor of WAB scores. Age-related changes in general cognition, as assessed by tests such as the MoCA^32,33^ or the Mini Mental State Exam (MMSE)^32,34^ are well-documented^8,9,35–37^, but the relationship between age and language-specific aspects of cognition is less clear. Some studies suggest that there is age-related decline in name retrieval, but no corresponding decline in retrieval of action-related words^38^. Similarly, age-related changes in language production often appear more notable than those in comprehension^37^. Research often suggests that the relationship between age and increased aphasia severity is driven by gray matter atrophy and potential cognitive reserve. However, as we found that age was an independent predictor of aphasia severity separate to both BrainGAP scores and atrophy (i.e., reduced gray matter volume), it is likely that other factors are also contributing to the relationship between age and aphasia severity. Other factors affect the integrity of non-lesioned brain tissue in older age and may be important for post-stroke outcomes, such as increased vascular risk factor burden and small vessel disease. Pathological white matter hyperintensities, a marker of small vessel disease, have been associated with larger ischemic lesion volumes^39,40^, poorer stroke outcomes^41,42^, and increased likelihood of post-stroke cognitive decline^43–46^. Future studies could incorporate measures of white matter hyperintensities, or other markers of small vessel disease, into models alongside age, gray matter volume (atrophy), and BrainGAP to investigate the contribution of small vessel disease.

An important aspect of the methodology in this paper was that each participant with aphasia had a different lesion volume and location, therefore the same ROIs were not used to estimate regional brain age for each participant. For each individual, we noted the number of ROIs used to estimate regional brain age, and we investigated the dispersion of the residuals to identify whether the number of ROIs affected the accuracy of the regional brain age prediction. There was a negative correlation between the number of ROIs used and the standard deviation of the residuals. This suggests that the number of ROIs used is linearly associated with the accuracy of the brain age estimation; however, as there was no significant relationship with the mean of the residuals, it suggests that there is not a tendency towards a higher or lower brain age estimation, the model is simply more accurate with more regions. However, in an effort to control for this, we included the number of ROIs used as an independent variable in the linear mixed effects models predicting behavior. It was a significant predictor for the spontaneous speech subtest. Therefore, the results should be interpreted with caution since the estimated brain age may be less accurate for participants with fewer ROIs remaining. The fact that the number of ROIs used was not a significant predictor for any other behavioral score suggests that the regional BrainGAP scores are predictors of behavior irrespective of how many ROIs were used to estimate brain age.

There are some limitations with the current study. For example, control participants were younger than participants with aphasia, although the range of ages for the both groups was broad (healthy controls 20−79 years, stroke participants 29−80 years). Also, in comparisons of regional brain age between controls and participants with stroke aphasia, only out-of-range controls could be used (i.e., those with an estimated whole brain age of >5% higher/lower than chronological age) as within-range controls were used to create the model which estimates regional brain age.

Furthermore, because all participants had large left-hemisphere lesions covering the majority of the language-specific regions, we are unable to draw any conclusions about specific lesioned regions for each participant, we can only draw conclusions about non-lesioned regions. Because of this, the participants in the current study were not an ideal model for language-specific aging as they all had stroke aphasia and therefore had large lesions in language regions of the brain. This enabled us to investigate the hypotheses relating to aging in domain-general brain regions only, meaning we cannot be sure that the brain age of language-specific regions are not also contributing to behavior. However, future studies could investigate aging specifically to language regions in different populations, possibly with right hemisphere lesions, or lesions elsewhere in the left hemisphere. Furthermore, throughout the manuscript we refer to any individual with a positive BrainGAP score as having premature brain aging, irrespective of the size of the gap. It is possible that having a small (positive or negative) BrainGAP is part of individual variability rather than premature brain aging. Future studies could investigate a potential critical threshold for classifying someone as having premature brain aging.

Finally, the data in this study is from a single timepoint following stroke therefore we are unable to investigate the brain age of participants prior to the stroke. It is possible that there are changes in brain age before stroke onset, possibly associated with cerebrovascular disease burden^47^. Therefore, it is not possible to make any conclusions about the rate of brain aging using the current data. Future studies could address this using longitudinal data.

In this study we used a brain age method to estimate global and regional brain age based on a normative cohort of healthy controls to investigate the relationship between regional brain aging and aphasia severity. For domain general regions, BrainGAP, gray matter volume, age, and lesion volume were significant predictors of aphasia severity and all WAB-R subtests, suggesting that isolated aging matters for behavior, and degradation to specific brain regions may be associated with behavioral outcomes.

## Methods

### Participants

#### Healthy controls

Healthy participants (*n* = 232) were part of the ABC@UofSC Repository^48^, an ongoing cross-sectional cohort study at the University of South Carolina. Participants were between 20 and 80 years of age and were at least proficient in English. Institutional Review Board Approval was obtained, followed by written informed consent provided by all participants at enrollment.

#### Stroke participants

Stroke participants (*n* = 89) were part of the POLAR (Predicting Outcomes of Language Rehabilitation) clinical trial (NCT0341678), with data collected at the Center for the Study of Aphasia Recovery (C-STAR) at the University of South Carolina (USC) and the Medical University of South Carolina (MUSC). Behavioral testing took place at research laboratories at USC and MUSC. ASHA-certified speech-language pathologists with experience working with individuals with aphasia administered all assessments and treatments. Aphasia type was determined based on the Western Aphasia Battery Revised (WAB-R^49^) classification consistent with published norms.

The following inclusion/exclusion criteria were applied. Inclusion criteria: (i) incurred a left-hemisphere ischemic or hemorrhagic stroke to the middle cerebral artery, (ii) had chronic aphasia (≥12 months post-stroke), (iii) were between 21 and 80 years of age, (iv) had spoken English as their primary language for at least 20 years, and (v) were able to provide written or verbal consent. Participants were excluded if they had (i) severely limited verbal output (as measured by a WAB-R^49^ Spontaneous Speech rating scale score of 0−1), (ii) severely impaired auditory comprehension (as measured by a WAB-R Auditory Comprehension rating scale score of 0−1), (iii) bilateral or cerebellar stroke, or (iv) or contra-indications to testing with magnetic resonance imaging (MRI). Individuals with multiple strokes were eligible if all lesions were confined to left supratentorial territory.

### Behavioral testing

As part of the POLAR protocol, stroke participants underwent extensive baseline language and neuropsychological testing, including the WAB-R^49^, as well as MRI at the time of enrollment^50^. For each participant, WAB-R aphasia quotient (AQ) was calculated along with the following WAB-R subscores: naming, spontaneous speech, repetition, and comprehension.

### Brain imaging

#### MRI data acquisition and preprocessing

Both healthy participants and participants with stroke aphasia underwent high-resolution T1- and T2-weighted neuroimaging on a Siemens Trio 3 T scanner equipped with a 12-channel (Trio configuration) or 20-channel (following upfit to Prisma configuration) head coil using the following parameters: T1-weighted imaging utilized an MP-RAGE sequence with 1 mm isotropic voxels, a 256 × 256 matrix size, a 9° flip angle, and a 92-slice sequence with repetition time (TR) = 2250 ms, inversion time (TI) = 925 ms, and echo time (TE) = 4.11 ms. T2-weighted scans were acquired using the same angulation and volume center as the T1 scan. This 3D T2-weighted SPACE sequence used a resolution of 1 mm^3^ was used with a field of view = 256 x 256 mm, 160 sagittal slices, variable degree flip angle, TR = 3200 ms, TE = 212 ms, x2 GRAPPA acceleration (80 reference lines).

For individuals with stroke aphasia, lesions were drawn onto each participant’s T2-weighted image by a neurologist (author LB) or trained study staff member (author RNN), both of whom were blinded to participant demographic information and WAB-R scores. Each participant’s T2 image was co-registered to their T1 image, and binary lesion maps were then spatially transformed into native T1 space using the resulting function. Resliced lesion maps were smoothed with a 3 mm full-width half maximum Gaussian kernel to remove sharp edges associated with hand drawing. Enantiomorphic segmentation-normalization was then conducted using the *nii_preprocess* pipeline (https://github.com/neurolabusc/nii_preprocess)^51^, a series of custom MATLAB-based (R2017b, TheMathWorks) scripts^51^ that leverage multiple best-of-breed programs (SPM12; Functional Imaging Laboratory, Wellcome Trust Centre for Neuroimaging, Institute of Neurology [www.fil.ion.ucl.ac.uk/spm], FSL v6.0.3^52^, ASLtbx [http://www.cnf.upenn.edu/~zewang/ASLtbx.php], and MRItrix [https://www.mrtrix.org/]) to normalize and process MRI data acquired from participants with lesioned brains. This included creation of a mirrored image of the right hemisphere, which was co-registered to the native T1 image. A chimeric image (i.e., a healed brain) was then created, based on the native T1 scan with the lesioned tissue replaced by tissue from the mirrored hemisphere^53^. SPM12’s unified segmentation-normalization^54^ warped this chimeric image to standard space, and the resulting spatial transform was applied to the native T1 scan as well as the lesion map and the T2/DWI image.

#### Brain age for healthy controls

Brain age estimation for control participants was performed on T1-weighted images using the BrainAgeR analysis pipeline (github.com/james-cole/brainageR) and the default protocol^23,55^. First, T1-weighted images were segmented into gray and white matter before being normalized using non-linear spatial registration^23^ and SPM12’s DARTEL toolbox^54^. The BrainAgeR analysis pipeline uses a customized version of FSL *slicesdir* to generate a directory of images and corresponding index.html files for quality controlling in a web browser (github.com/james-cole/brainageR). These probabilistic tissue maps were visually inspected by an expert neurologist (author LB) to ensure quality of the segmentation. The cerebrospinal fluid tissue was removed, and the gray and white matter probabilistic tissues were vectorized, concatenated, and subjected to a principal components analysis to reduce dimensionality. The components explaining the top 80% of the variance were used for brain age prediction. A machine-learning algorithm using a pretrained Gaussian regression model implemented in the R package *Kernlab* was used to estimate brain age. This pretrained model was based on scans of 3377 healthy individuals from 7 publicly available datasets and tested on 611 different scans of healthy individuals aged between 18 and 90 years^23^. See Cole and colleagues for more detail on the BrainAgeR pipeline^23^. It is important to note that this is the only time throughout the paper where a global brain age score is used.

To create accurate baseline models of gray matter volume and age, only controls with an estimated brain age within 5% of their chronological age were used to create future linear models (*n* = 126). For example, data from Participant A with an estimated brain age of 82 and a chronological age of 80 would be used in the models, whereas data from Participant B with an estimated brain age of 85 and a chronological age of 80 would not be used in the linear model. Included control participants will be referred to as within-range controls in future descriptions (*n* = 126). Those excluded at this step are henceforth referred to as out-of-range controls (*n* = 106).

#### Gray Matter Volume

Both total gray matter volume and gray matter volume of each region of interest (ROI) in the Johns Hopkins University (JHU) atlas were also calculated for all controls (*n* = 216) and participants with aphasia (*n* = 89) using the Computational Anatomy Toolbox (CAT12) for SPM12 (Wellcome Department of Cognitive Neurology). Voxel-based morphometry was used to estimate the volume of gray matter using the following steps and default parameters: (1) spatial registration to a reference brain template, (2) tissue classification (segmentation) into gray matter, white matter, and cerebrospinal fluid (CSF), then (3) bias correction of intensity non-uniformities^54^. For each participant, gray matter volume was divided by total intracranial volume to account for variation in brain volume.

#### Classifying regions of the brain

**Fig. 5:**
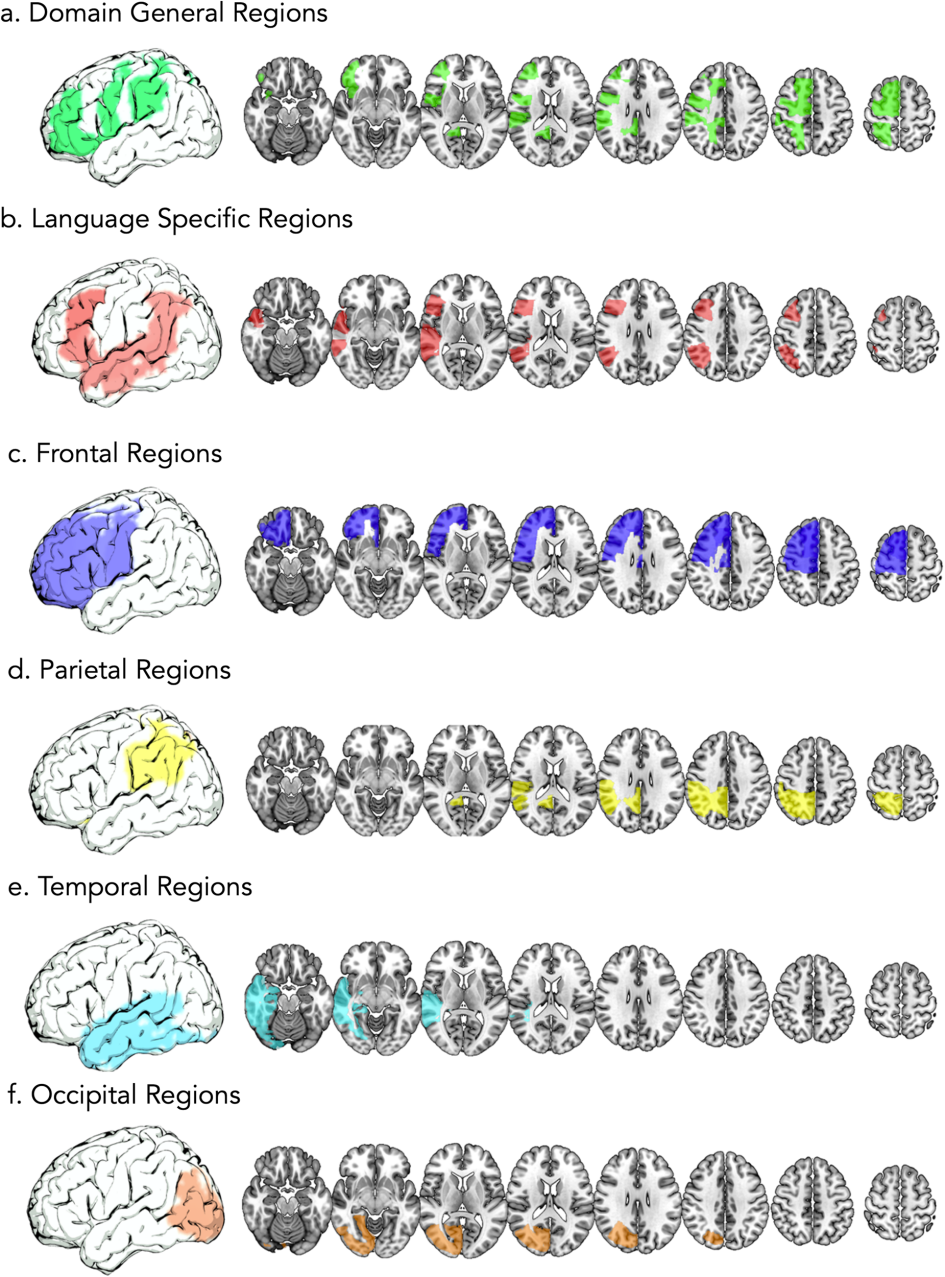
Groupings of ROIs into brain regions. Groupings of ROIs from the JHU atlas into brain regions. **a** shows domain general regions, (**b**) language specific regions, (**c**) frontal regions, (**d**) parietal regions, (**e**) temporal regions, and (**f**) occipital regions.

ROIs (189) from the JHU atlas were grouped into different brain regions, see Fig. 5. The following groups were created; ROIs within the left hemisphere (94 ROIs), right hemisphere (94 ROIs), domain general regions (16 ROIs: left: 8, right: 8), language-specific regions (18 ROIs, left: 9, right: 9), ROIs in the frontal lobe (32 ROIs, left: 16, right: 16), temporal lobe (26 ROIs, left: 13, right: 13), parietal lobe (12 ROIs, left:6, right: 6), and occipital lobe (10 ROIs, left: 5, right: 5). A full breakdown of which ROIs were in each region can be found in Supplementary Data 2. Graphs to show gray matter volume in controls and proportion of lesion in each region can be found in Supplementary Fig. 1. In all subsequent text, ‘ROIs’ will refer to areas in the JHU atlas (e.g., left superior frontal gyrus, right superior frontal gyrus, etc.), while ‘regions’ will refer to these brain areas comprised of ROIs (e.g., domain general regions, language-specific regions).

#### Calculating regional brain age in participants with Aphasia

For each participant with aphasia, overlap with the lesion and ROIs in the JHU atlas was calculated to identify how much of each ROI was lesioned. For each region (e.g., language-specific region), all ROIs with at least 99% spared by the lesion were identified. Using those identified ROIs, for each participant with aphasia, a multiple linear regression was calculated between the intact ROIs and age in the within-range control participants to estimate the multivariate relationship between the gray matter volume in these ROIs and regional brain age. Using this model, it was possible to estimate the regional brain age of the individual with aphasia (i.e., the combined brain age of the ROIs used in the model e.g., left domain general regions). For example, for left domain-general regions, if Participant A has a lesion covering all left domain-general ROIs except superior frontal gyrus (SFG) and middle frontal gyrus (MFG), in the within-range control participants a multiple linear regression would be calculated where the dependent variable was their estimated brain age from the BrainAgeR pipeline, and the independent variables were the gray matter volume of left SFG and left MFG to generate a multivariate model. Then, the volume of left SFG and left MFG in Participant A could be entered into the multivariate model to give an estimate of their left domain-general regional brain age. For Participant B, who may have no lesioned left domain-general ROIs, the volume of all 8 left domain-general ROIs would be used to generate the multivariate model in the within-range controls. Then, the gray matter volume of all of Participant B’s 8 left domain-general ROIs would be entered into the model to estimate their left domain-general regional brain age.

**Fig. 6:**
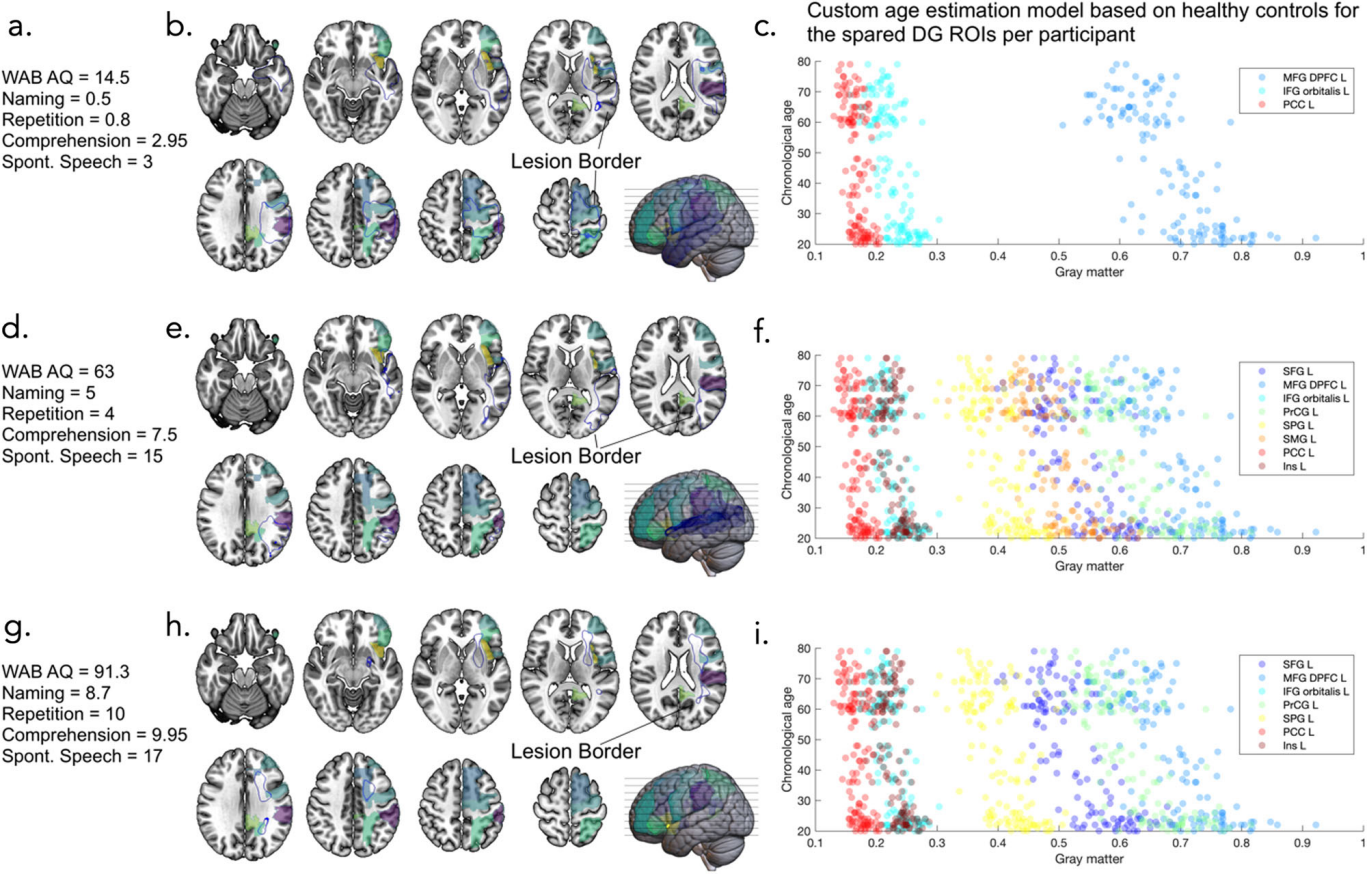
Brain age estimations for domain-general regions. Brain age estimation of domain-general regions for 3 example participants to highlight the use of different ROIs for each individual brain age estimation based on which ROIs were spared by the lesion. show the first participant, where (**a**) gives an outline of their behavioral scores, (**b**) shows an outline of their lesion (blue line) alongside the left domain general ROIs, and (**c**) shows the custom age estimation model based on healthy controls for the spared domain-general ROIs for this participant (note they are different for each participant based on which ROIs are lesioned). Similarly, (**d**, **g**) show behavioral scores for 2 other participants, (**e**, **h**) show brain maps of the overlap between their lesions and the domain general ROIs, and (**f**, **i**) show their custom age estimation models. Note that figures are in neurological orientation so the left (lesioned) hemisphere is shown on the right. WAB AQ Western Aphasia Battery Aphasia Quotient, Spont. Speech Spontaneous Speech, DG domain general, ROIs regions of interest, MFG DPFC L middle frontal gyrus dorsal prefrontal cortex left, IFG orbitalis L inferior frontal gyrus orbitalis left, PCC L posterior cingulate gyrus left, SFG L superior frontal gyrus left, PrCG L precentral gyrus left, SMG L supramarginal gyrus left, Ins L insular left.

It is important to highlight that each participant with aphasia has a different lesion volume and location, meaning that different ROIs were affected by the lesion. As each linear equation is derived from the ROIs in each region which are spared, the same ROIs are not used for each participant. As described above, Participant A may have only had 2 spared (i.e., non-lesioned) left domain-general ROIs (therefore 6 lesioned) so for this participant, only 2 ROIs would be used to create the linear model in control participants and then to estimate regional brain age of Participant A. Conversely, Participant B may have had no lesioned left domain-general ROIs, so all 8 left domain-general ROIs would be used to create the linear model in control participants and then to estimate the regional brain age of Participant B. For participants with no spared language-specific ROIs, no linear model was created in control participants, and this individual was excluded from the future statistics using language-specific ROIs. However, if ROIs in the domain-general region were spared, this individual would be used for domain-general region statistics, see Fig. 6. For each participant, the number of ROIs used to generate each regional linear regression model was recorded, then the dispersion of the residuals was investigated to determine whether the number of ROIs used in the prediction affected the accuracy of the brain age prediction (i.e., if the left domain-general regional brain age prediction would be significantly better or worse for Participant A who had a lesion which covered 6 of 8 ROIs in the left domain-general region versus Participant B who had a lesion covering no ROIs in the left domain-general region).

For each set of regions (i.e., left hemisphere gray matter, language-specific regions, domain-general regions, etc.), brain age was estimated for each participant. The brain age gap was also calculated using the following equation: brain age gap = brain age – chronological age. This will henceforth be referred to as BrainGAP, where a positive BrainGAP suggests that estimated brain age is older than chronological age (i.e., premature brain aging). It is important to note that throughout the manuscript, we refer to any individual with a positive BrainGAP as having premature brain aging, irrespective of the size of the gap.

#### Calculating regional brain age in out-of-range controls

Using the same method as described above for participants with aphasia, regional brain age was calculated for out-of-range controls (*n* = 106: healthy participants whose whole brain age estimated using the BrainAgeR pipeline was more than 5% above/below their chronological age). We calculated this is to compare regional brain age in healthy control participants compared to stroke participants, and it was not possible to calculate this for in-range controls as they were used to create the estimation model and therefore regional brain age estimations could not be generated for this group. It is therefore important to note that all ROIs were included for each region as these participants had no lesioned tissue.

### Statistics

#### Statistics and reproducibility

Statistical analyses were conducted using Matlab (R2017b, TheMathWorks). All participants were included in statistical analyses where described (PWA: 89 participants, within-range controls: 126 participants, out-of-range controls: 106 participants). All scripts used for analysis will be made available on Dr Bonilha and Dr Rorden’s Github.

#### Mixed effects ANCOVA between hemispheres and controls vs stroke participants

To identify if there were differences in BrainGAP between hemispheres, and between out-of-range controls and stroke participants, a two-way 2 (group: control or stroke aphasia) x 2 (hemisphere: left or right) ANCOVA was conducted with BrainGAP in the domain general ROIs as the dependent variable and the following as covariates: chronological age, years of education, sex and handedness.

#### Independent sample T-tests between controls and stroke participants

To investigate whether there was a statistical difference in the age of control participants and participants with aphasia, independent sample *t* tests were conducted.

#### Correlation analysis between regional brain age and gray matter volume

To investigate whether there was a significant relationship between regional brain age and regional gray matter volume (accounting for total intracranial volume) in participants with aphasia, Pearson correlations were conducted for every region (i.e., left hemisphere, right hemisphere, left language-specific regions, right language-specific regions, left domain-general regions, right domain-general regions, left frontal regions, right frontal regions, left temporal regions, right temporal regions, left parietal regions, right parietal regions, left occipital regions and right occipital regions).

#### Number of ROIs used in the linear model

We investigated the dispersion of the residuals to determine whether the number of ROIs used to estimate regional brain age affected the accuracy of the brain age prediction (i.e., if the domain-general regional brain age prediction would be worse for Participant A who had a lesion which covered 2 ROIs in the domain-general region vs Participant B who had a lesion covering 0 ROIs in the domain-general region). To do this, we ran correlations between the number of ROIs used and both the standard deviation of the residuals, and the mean of the residuals. For these analyses, a correlation coefficient closer to 0 would indicate that the number of ROIs used in the model did not strongly influence the accuracy of the brain age prediction. A strong negative correlation might suggest that the brain age prediction was less accurate when a participant had less intact ROIs, while a strong positive correlation suggests that the brain age predictions were more accurate with fewer ROIs.

#### Multiple linear regression analysis between regional brain age and behavior

We evaluated the relationship between regional brain age and aphasia severity (WAB AQ and subscores) using multiple linear regression models in which the behavioral variable was set as the dependent variable, and the following variables were used as independent variables: BrainGAP, atrophy (average gray matter volume), lesion volume, participant age, and number of ROIs used in the linear regression model. Given that the WAB AQ and WAB subscores are likely correlated, we adjusted alpha levels of the statistical tests to account for multiple comparisons and to reduce the likelihood of Type 1 errors (0.05/5 = 0.01).

### Study approval

This study was approved by the University of South Carolina IRB Committee. All participants gave informed consent for study participant in accordance with the Declaration of Helsinki. All ethical regulations relevant to human research participants were followed.

### Reporting summary

Further information on research design is available in the Nature Portfolio Reporting Summary linked to this article.

### Supplementary information

Supplementary Material

Description of Additional Supplementary Files

Supplementary Data 1

Supplementary Data 2

Reporting Summary

## Data Availability

Source data will be shared upon reasonable request to the corresponding author. Due to the limitations of our ethics, a request is deemed as reasonable if it does not require identifiable information relating to the participants. Data used to create graphs are included in Supplementary Data 1.

## Code availability

Preprocessing scripts are available at the following repository: 10.5281/ZENODO.4027711. All scripts used for analysis will be made available on Dr Bonilha and Dr Rorden’s Github.
