# Supplementary material for: Regional brain aging: premature aging of the domain general system predicts aphasia severity
Source: Commun Biol. 2024 Jun 11;7:718. doi: 10.1038/s42003-024-06211-8 (PMC11167062; doi:10.1038/s42003-024-06211-8)
Supplement: Supplementary file 1 — Supplementary Material [file 42003_2024_6211_MOESM1_ESM.pdf]

Supplementary Table 1. Demographic information of participants with aphasia

**Demographic Variables**	**Value**	**T-Test Results**	
		**Compared to Within-Range Controls**	**Compared to Out-of-Range Controls**
Stroke age, mean (SD)	56.69 (11.94)		
Test age, mean (SD), range	60.60 (11.27), 29-80	$t = -7.082$, $p < 0.001$*	$t = 7.381$, $p < 0.001$*
Education, mean (SD)	15.49 (2.28)	$t = 3.079$, $p = 0.002$*	$t = 0.229$, $p = 0.820$
Sex (males:females), count	54:35	$t = -4.274$, $-p < 0.001$*	$t = -6.262$, $p < 0.001$*
(percentage)	(60.67%:39.33%)		
Handedness (left:right), count	9:79	$t = 1.629$, $p = 0.105$	$t = 1.984$, $p = 0.048$*
(percentage)	(10.23%:89.77%)		
Months post-stroke, mean (SD)	46.35 (47.57)		
Aphasia Fluency (fluent:nonfluent),	45:44		
count (percentage)	(50.56%:49.44%)		
Western Aphasia Battery	59.15 (22.71)		
Aphasia Quotient, mean (SD)			
Aphasia Types count (percentage)			
Anomic	25 (28.09%)		
Broca's	41 (46.07%)		
Conduction	12 (13.48%)		
Global	4 (4.49%)		
Transcortical Motor	1 (1.12%)		
Wernicke's	6 (6.74%)		

Supplementary Table 2. Demographic information of within-range controls (n = 126) and out-of-range controls (n = 106).

**Within-Range Controls (*n* = 126)**	
**Demographic Variables**	**Value**
Test age, mean (SD), range	46.13 (19.93), 20-79
Sex (males:females), count (percentage)	37:89 (29.37%, 70.63%)
Education (years)	16.37 (1.60)
Handedness (left:right:ambidextrous), count (percentage)	8:113:5 (6.35%, 89.68%, 3.97%)
MoCA Score, mean (SD)	27.18 (2.43)
**Out-of-Range Controls (*n* = 106)**	
**Demographic Variables**	**Value**
Test age, mean (SD)	46.80 (17.08), 20-79
Sex (males:females), count (percentage)	87:19 (82.08%, 17.92%)
Education (years)	15.52 (2.20)
Handedness (left:right:ambidextrous), count (percentage)	5:97:4 (4.7%, 91.51%, 3.77%)
MoCA Score, mean (SD)	27.50 (2.68)

Supplementary Table 3. Summary of the multiple linear regression analysis using left domain-general regions. Each row summarizes a separate linear regression model, with the behavioral test as the dependent variable, and BrainGAP, gray matter volume, lesion volume, age, and number of ROIs as the independent variables. Values indicate *t*-values on the top and *p*-values underneath. Significant values are indicated in **bold.**

Behavioral Test	BrainGAP	Gray Matter Volume	Lesion Volume	Age	Nu
WAB AQ	**-2.71**<br>**0.008**	**-2.67**<br>**0.009**	**-3.43**<br>**< 0.001**	**-3.47**<br>**< 0.001**	
WAB Naming	-2.11<br>0.038	-2.12<br>0.037	**-3.23**<br>**< 0.001**	**-3.05**<br>**< 0.001**	
WAB Repetition	-2.04<br>0.045	-2.17<br>0.033	**-3.09**<br>**0.003**	**-2.90**<br>**0.005**	
WAB Comprehension	**-3.34**<br>**0.001**	**-3.38**<br>**0.001**	**-4.56**<br>**< 0.001**	**-2.91**<br>**0.005**	
WAB Spontaneous Speech	-2.36<br>0.021	-2.15<br>0.034	-2.39<br>0.019	**-3.11**<br>**0.003**	

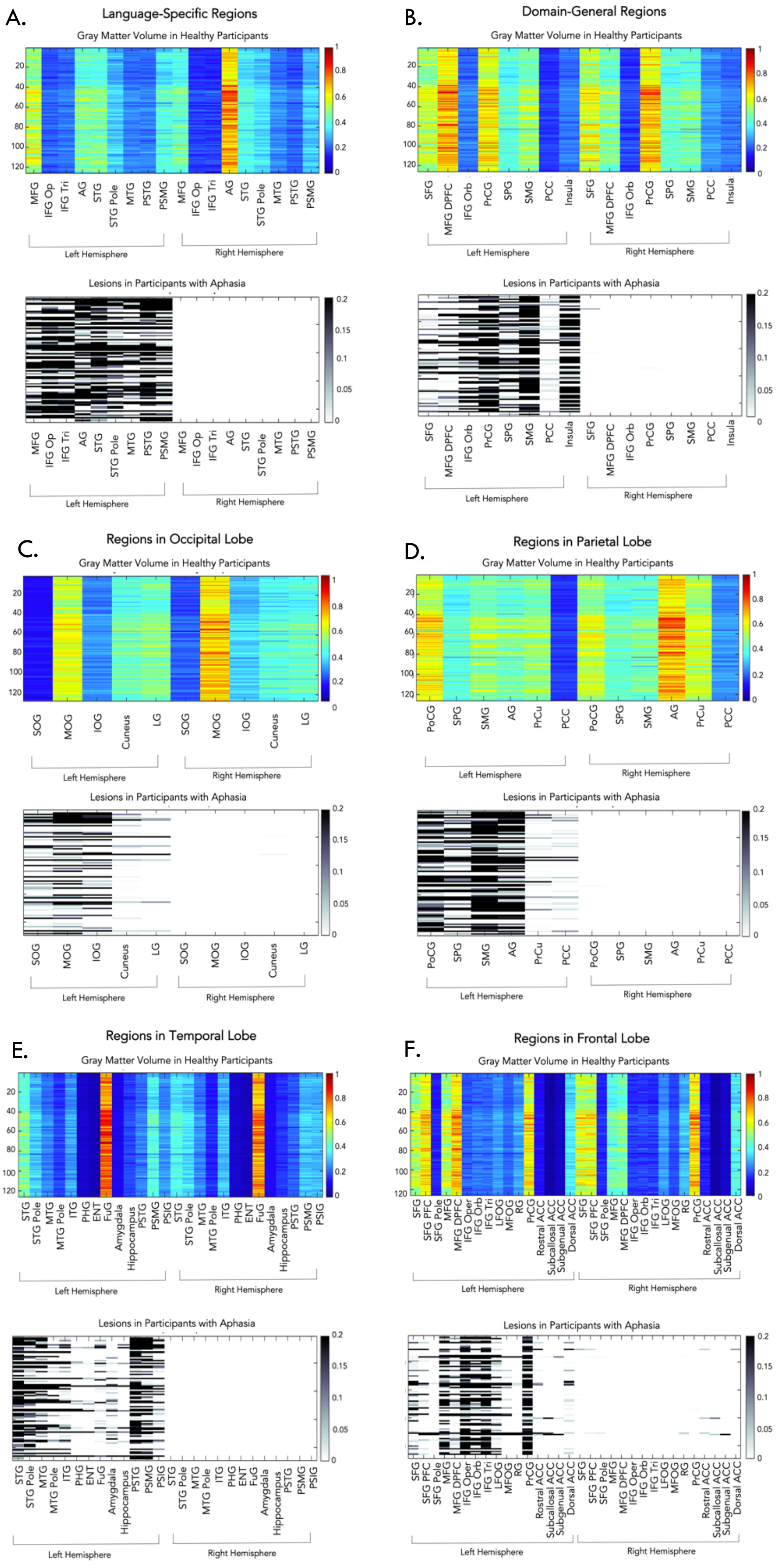

Supplementary Figure 1. Figures to show gray matter volume in healthy participants (upper color graph) and proportion of lesion to the corresponding ROIs in participants with aphasia (lower black and white graph). In all graphs, each line represents an individual participant. In the colored graphs, the scale shows probabilistic gray matter volume from VBM. On the black and white graphs, the scale shows the proportion of each ROI that has been lesioned. (A) shows language-specific regions, (B) shows domain-general, (C) occipital lobe, (D) parietal lobe, (E) temporal lobe, (F) frontal lobe.
