# Supplementary material for: Regional brain aging: premature aging of the domain general system predicts aphasia severity
Source: Commun Biol. 2024 Jun 11;7:718. doi: 10.1038/s42003-024-06211-8 (PMC11167062; doi:10.1038/s42003-024-06211-8)
Supplement: Supplementary file 2 — Description of Additional Supplementary Files [file 42003_2024_6211_MOESM2_ESM.pdf]

Description of Additional Supplementary Files

File name: Supplementary Data 1

Description: Data which support the graphs in the manuscript, including regional brain ages, regional proportional brain ages, and regional gray matter volumes.

File name: Supplementary Data 2

Description: A breakdown of which regions of interest from the Johns Hopkins atlas were in each brain region.
