# Supplementary material for: Regional brain aging: premature aging of the domain general system predicts aphasia severity
Source: Commun Biol. 2024 Jun 11;7:718. doi: 10.1038/s42003-024-06211-8 (PMC11167062; doi:10.1038/s42003-024-06211-8)
Supplement: Supplementary file 4 — Supplementary Data 2 [file 42003_2024_6211_MOESM4_ESM.docx]

| **Supplementary Data 2.** A breakdown of which regions of interest (ROI) from the Johns Hopkins atlas were in each brain region | | | | |
| --- | --- | --- | --- | --- |
| ROI Number | Abbreviated Name | Domain-General (DG) or Language-Specific (LS) | Hemisphere (Left: L, Right: R) | Lobe |
| 1 | SFG_L | DG | L | Frontal |
| 2 | SFG_R | DG | R | Frontal |
| 3 | SFG_PFC_L | Other | L | Frontal |
| 4 | SFG_PFC_R | Other | R | Frontal |
| 5 | SFG_pole_L | Other | L | Frontal |
| 6 | SFG_pole_R | Other | R | Frontal |
| 7 | MFG_L | LS | L | Frontal |
| 8 | MFG_R | LS | R | Frontal |
| 9 | MFG_DPFC_L | DG | L | Frontal |
| 10 | MFG_DPFC_R | DG | R | Frontal |
| 11 | IFG_opercularis_L | LS | L | Frontal |
| 12 | IFG_opercularis_R | LS | R | Frontal |
| 13 | IFG_orbitalis_L | DG | L | Frontal |
| 14 | IFG_orbitalis_R | DG | R | Frontal |
| 15 | IFG_triangularis_L | LS | L | Frontal |
| 16 | IFG_triangularis_R | LS | R | Frontal |
| 17 | LFOG_L | Other | L | Frontal |
| 18 | LFOG_R | Other | R | Frontal |
| 19 | MFOG_L | Other | L | Frontal |
| 20 | MFOG_R | Other | R | Frontal |
| 21 | RG_L | Other | L | Frontal |
| 22 | RG_R | Other | R | Frontal |
| 23 | PoCG_L | Other | L | Parietal |
| 24 | PoCG_R | Other | R | Parietal |
| 25 | PrCG_L | DG | L | Frontal |
| 26 | PrCG_R | DG | R | Frontal |
| 27 | SPG_L | DG | L | Parietal |
| 28 | SPG_R | DG | R | Parietal |
| 29 | SMG_L | DG | L | Parietal |
| 30 | SMG_R | DG | R | Parietal |
| 31 | AG_L | LS | L | Parietal |
| 32 | AG_R | LS | R | Parietal |
| 33 | PrCu_L | Other | L | Parietal |
| 34 | PrCu_R | Other | R | Parietal |
| 35 | STG_L | LS | L | Temporal |
| 36 | STG_R | LS | R | Temporal |
| 37 | STG_L_pole | LS | L | Temporal |
| 38 | STG_R_pole | LS | R | Temporal |
| 39 | MTG_L | LS | L | Temporal |
| 40 | MTG_R | LS | R | Temporal |
| 41 | MTG_L_pole | Other | L | Temporal |
| 42 | MTG_R_pole | Other | R | Temporal |
| 43 | ITG_L | Other | L | Temporal |
| 44 | ITG_R | Other | R | Temporal |
| 45 | PHG_L | Other | L | Temporal |
| 46 | PHG_R | Other | R | Temporal |
| 47 | ENT_L | Other | L | Temporal |
| 48 | ENT_R | Other | R | Temporal |
| 49 | FuG_L | Other | L | Temporal |
| 50 | FuG_R | Other | R | Temporal |
| 51 | SOG_L | Other | L | Occipital |
| 52 | SOG_R | Other | R | Occipital |
| 53 | MOG_L | Other | L | Occipital |
| 54 | MOG_R | Other | R | Occipital |
| 55 | IOG_L | Other | L | Occipital |
| 56 | IOG_R | Other | R | Occipital |
| 57 | Cu_L | Other | L | Occipital |
| 58 | Cu_R | Other | R | Occipital |
| 59 | LG_L | Other | L | Occipital |
| 60 | LG_R | Other | R | Occipital |
| 61 | rostral_ACC_L | Other | L | Frontal |
| 62 | rostral_ACC_R | Other | R | Frontal |
| 63 | subcallosal_ACC_L | Other | L | Frontal |
| 64 | subcallosal_ACC_R | Other | R | Frontal |
| 65 | subgenual_ACC_L | Other | L | Frontal |
| 66 | subgenual_ACC_R | Other | R | Frontal |
| 67 | dorsal_ACC_L | Other | L | Frontal |
| 68 | dorsal_ACC_R | Other | R | Frontal |
| 69 | PCC_L | DG | L | Parietal |
| 70 | PCC_R | DG | R | Parietal |
| 71 | Ins_L | DG | L | Insula |
| 72 | Ins_R | DG | R | Insula |
| 73 | Amyg_L | Other | L | Temporal |
| 74 | Amyg_R | Other | R | Temporal |
| 75 | Hippo_L | Other | L | Temporal |
| 76 | Hippo_R | Other | R | Temporal |
| 77 | Caud_L | Other | L | Basal Nuclei |
| 78 | Caud_R | Other | R | Basal Nuclei |
| 79 | Put_L | Other | L | Basal Nuclei |
| 80 | Put_R | Other | R | Basal Nuclei |
| 81 | GP_L | Other | L | Basal Nuclei |
| 82 | GP_R | Other | R | Basal Nuclei |
| 83 | Thal_L | Other | L | Basal Nuclei |
| 84 | Thal_R | Other | R | Basal Nuclei |
| 85 | Hypothalamus_L | Other | L | Subcortical |
| 86 | Hypothalamus_R | Other | R | Subcortical |
| 87 | Mynert_L | Other | L | Subcortical |
| 88 | Mynert_R | Other | R | Subcortical |
| 89 | NucAccumbens_L | Other | L | Subcortical |
| 90 | NucAccumbens_R | Other | R | Subcortical |
| 91 | RedNc_L | Other | L | Subcortical |
| 92 | RedNc_R | Other | R | Subcortical |
| 93 | Snigra_L | Other | L | Subcortical |
| 94 | Snigra_R | Other | R | Subcortical |
| 95 | cerebellum_R | Other | L | Cerebellum |
| 96 | cerebellum_L | Other | R | Cerebellum |
| 97 | CP_L | Other | L | Subcortical |
| 98 | CP_R | Other | R | Subcortical |
| 99 | Midbrain_L | Other | L | Subcortical |
| 100 | Midbrain_R | Other | R | Subcortical |
| 101 | CST_L | Other | L | None |
| 102 | CST_R | Other | R | None |
| 103 | SCP_L | Other | L | None |
| 104 | SCP_R | Other | R | None |
| 105 | MCP_L | Other | L | None |
| 106 | MCP_R | Other | R | None |
| 107 | PCT_L | Other | L | None |
| 108 | PCT_R | Other | R | None |
| 109 | ICP_L | Other | L | None |
| 110 | ICP_R | Other | R | None |
| 111 | ML_L | Other | L | None |
| 112 | ML_R | Other | R | None |
| 113 | Pons_L | Other | L | None |
| 114 | Pons_R | Other | R | None |
| 115 | Medulla_L | Other | L | None |
| 116 | Medulla_R | Other | R | None |
| 117 | ACR_L | Other | L | None |
| 118 | ACR_R | Other | R | None |
| 119 | SCR_L | Other | L | None |
| 120 | SCR_R | Other | R | None |
| 121 | PCR_L | Other | L | None |
| 122 | PCR_R | Other | R | None |
| 123 | GCC_L | Other | L | None |
| 124 | GCC_R | Other | R | None |
| 125 | BCC_L | Other | L | None |
| 126 | BCC_R | Other | R | None |
| 127 | SCC_L | Other | L | None |
| 128 | SCC_R | Other | R | None |
| 129 | TAP_L | Other | L | None |
| 130 | TAP_R | Other | R | None |
| 131 | ALIC_L | Other | L | None |
| 132 | ALIC_R | Other | R | None |
| 133 | PLIC_L | Other | L | None |
| 134 | PLIC_R | Other | R | None |
| 135 | RLIC_L | Other | L | None |
| 136 | RLIC_R | Other | R | None |
| 137 | EC_L | Other | L | None |
| 138 | EC_R | Other | R | None |
| 139 | CGC_L | Other | L | None |
| 140 | CGC_R | Other | R | None |
| 141 | CGH_L | Other | L | None |
| 142 | CGH_R | Other | R | None |
| 143 | Fx/ST_L | Other | L | None |
| 144 | Fx/ST_R | Other | R | None |
| 145 | Fx_L | Other | L | None |
| 146 | Fx_R | Other | R | None |
| 147 | IFO_L | Other | L | None |
| 148 | IFO_R | Other | R | None |
| 149 | PTR_L | Other | L | None |
| 150 | PTR_R | Other | R | None |
| 151 | SS_L | Other | L | None |
| 152 | SS_R | Other | R | None |
| 153 | SFO_L | Other | L | None |
| 154 | SFO_R | Other | R | None |
| 155 | SLF_L | Other | L | None |
| 156 | SLF_R | Other | R | None |
| 157 | UNC_L | Other | L | None |
| 158 | UNC_R | Other | R | None |
| 159 | AnsaLenticularis_L | Other | L | None |
| 160 | AnsaLenticularis_R | Other | R | None |
| 161 | AnteriorCom_L | Other | L | None |
| 162 | AnteriorCom_R | Other | R | None |
| 163 | LenticularFasc_L | Other | L | None |
| 164 | LenticularFasc_R | Other | R | None |
| 165 | OlfactoryRadiation_L | Other | L | None |
| 166 | OlfactoryRadiation_R | Other | R | None |
| 167 | Mammillary_L | Other | L | Subcortical |
| 168 | Mammillary_R | Other | R | Subcortical |
| 169 | OpticTract_L | Other | L | None |
| 170 | OpticTract_R | Other | R | None |
| 171 | LV_frontal_L | Other | L | CSF |
| 172 | LV_body_L | Other | L | CSF |
| 173 | LV_atrium_L | Other | L | CSF |
| 174 | LV_occipital_L | Other | L | CSF |
| 175 | LV_temporal_L | Other | L | CSF |
| 176 | LV_frontal_R | Other | R | CSF |
| 177 | LV_body_R | Other | R | CSF |
| 178 | LV_atrium_R | Other | R | CSF |
| 179 | LV_occipital_R | Other | R | CSF |
| 180 | LV_temporal_R | Other | R | CSF |
| 181 | III_and_IV_ventricle | Other | R | CSF |
| 182 | PIns_L | Other | L | Insula |
| 183 | PIns_R | Other |  | Insula |
| 184 | PSTG_L | LS | L | Temporal |
| 185 | PSTG_R | LS | R | Temporal |
| 186 | PSMG_L | LS | L | Temporal |
| 187 | PSMG_R | LS | R | Temporal |
| 188 | PSIG_L | Other | L | Temporal |
| 189 | PSIG_R | Other | R | Temporal |
